# High Ecophysiological Plasticity of *Desmarestia aculeata* (Phaeophyceae) Present in an Arctic Fjord under Varying Salinity and Irradiance Conditions

**DOI:** 10.3390/biology11101499

**Published:** 2022-10-13

**Authors:** Johanna Marambio, Nora Diehl, Kai Bischof

**Affiliations:** 1 Marine Botany, University of Bremen, Leobner Str. NW2, 28359 Bremen, Germany; 2 Alfred Wegener Institute for Polar and Marine Research, Functional Ecology, 27570 Bremerhaven, Germany; 3 Laboratory of Antarctic and Sub-Antarctic Marine Ecosystems (LEMAS), Department of Sciences, University of Magallanes, Punta Arenas 6200000, Chile; 4 Cape Horn International Center (CHIC), University of Magallanes, Punta Arenas 6200000, Chile; 5 Center for Marine Environmental Sciences, University of Bremen, 28359 Bremen, Germany

**Keywords:** Arctic, mannitol, acclimation, *F*_v_/*F*_m_, pigments

## Abstract

**Simple Summary:**

The Arctic region has been affected by rising temperatures, directly affecting the organisms living there. One of the organisms that inhabit this area is the seaweed *Desmarestia aculeata* (Phaeophyceae), widely distributed in the North Atlantic. It is exposed to the high Arctic light regime and fluctuating salinity conditions from glacial and terrestrial run-off. Despite its abundance, little is known about *D. aculeata* and how environmental drivers will affect it in a future altered by climate change. During the summer of 2019, *D. aculeata* was collected in Kongsfjorden, Svalbard (78.9° N, 11.9° E) to investigate its physiological and biochemical responses to salinities of 34, 28, and 18, and daily cycles of irradiance (50–500 μmol photons m^−2^s^−1^) at 0 °C over 21 days. Photosynthetic parameters and high pigment concentrations show how this species has an effective acclimation to irradiance changes, being unaffected by low salinity. The high concentration of antioxidant phlorotannins at low salinity show how *D. aculeata* can regulate its daily cycle despite the hyposaline conditions. Salinity and light are interacting factors in the acclimation process. Our work shows the high plasticity of *D. aculeata*, such that the species will probably be able to tolerate future changes in the Arctic.

**Abstract:**

The seaweed *Desmarestia aculeata* (Phaeophyceae) is distributed in the temperate zone of the North Atlantic up to the Arctic, where it is exposed to a high Arctic light regime and fluctuating salinity conditions resulting from glacial and terrestrial run-off. Information on how this species is able to thrive under current and future Arctic conditions is scarce. During the Arctic summer of 2019, *D. aculeata* was collected in Kongsfjorden, Svalbard (78.9° N, 11.9° E) to investigate its physiological and biochemical responses to variations in salinity (salinities: 34, 28 and 18) and daily cycles of irradiance (50–500 μmol photons m^−2^s^−1^) at 0 °C over 21 days. The species revealed effective short-term acclimation to both abiotic drivers. Maximal quantum yield of PSII (*F*_v_/*F*_m_) fluctuated with the light cycle at a salinity of 34, while the maximum relative electron transport rate (rETRmax) significantly differed between salinities of 28 and 18. Chlorophyll *a* and β-Carotene remained at high concentrations in all treatments showing pronounced acclimation during the experiment. High mannitol concentrations were measured throughout the experiment, while phlorotannins were high at low salinity. Hyposalinity and light are interacting drivers of the physiological and biochemical acclimation process for *D. aculeata*. Our experiment highlights the high ecophysiological plasticity of *D. aculeata*, suggesting that the species will likely be capable of withstanding future habitat changes in the Arctic.

## 1. Introduction

Global warming generates a series of changes in Arctic marine biota, also strongly affecting the Svalbard Archipelago [1]. Marine benthic communities are constantly interacting with changes in environmental factors, especially in the intertidal zone. Increasing temperatures in the surface waters of Arctic fjords [2] result in changes in the thickness and extent of sea ice [3]. Furthermore, elevated atmospheric temperatures will increase the meltwater inflow to the fjords, altering both salinity and irradiance conditions in the water column [4]. These changes directly affect marine benthic communities, which have long been the focus of studies in Kongsfjorden, Svalbard [5]. There, habitat-providing ecosystem engineers, such as marine seaweeds, are of high ecological importance [6,7] and are key primary producers [8]. More than 197 species of seaweed have been described for the Svalbard region [9]. Among them, the brown alga *Desmarestia aculeata* has been shown to host a particularly diverse associated fauna comprised of 36 invertebrate species [6].

There are four possible responses of benthic communities to environmental perturbations: acclimation, adaptation, migration or death [10,11]. Therefore, only species with high ecophysiological plasticity are likely to prevail in shifting marine environments. *Desmarestia aculeata* can adjust pigmentation and photosynthetic responses to pronounced environmental variation throughout the summer period in Kongsfjorden, decreasing chlorophyll *a* concentration in the months of highest irradiance [12,13] *D. aculeata* has a wide distribution in the North Atlantic [14], even extensively inhabiting the Arctic coastal zone [15,16]. It is frequently found in association with its congener species *D. viridis* [17,18]. The species is also commonly found attached to rocks and as an epiphyte on other brown seaweeds, such as *Saccharina latissima* or *Laminaria hyperborea* [19,20], and forms extensive submarine meadows in the shallow subtidal and intertidal zones during the summer [17].

Previous studies in *D. aculeata* have described the lack of gene regulation under variations in temperature and carbon dioxide (CO_2_) levels [21], presumably resulting in high energy costs for maintenance. Furthermore, nutrient assimilation does not seem to be affected by nutrient enrichment during the summer season, thus presenting a plastic response towards this factor [22]. The effect of irradiance and temperature, and the interaction between these factors, on photosynthetic parameters (α, rETRmax or *E*k) and biochemical analysis (pigment and antioxidant analysis) has been previously reported for *D. aculeata* [12,23,24]. In this context, temperature defines the distribution range of *D. aculeata*, due to its wide tolerance range of 0–20 °C [25]. The effect of other factors, such as hyposalinity, has been previously described as having negative effects on algae, e.g., *P. palmata* [26] or *Alaria esculenta* [27]. However, other species, such as *Laminaria digitata* or *L. solidungula*, show a high tolerance to hyposaline conditions [28]. In general, few studies have tested the acclimation responses of *D. aculeata* to multiple, and presumably interacting, abiotic factors, particularly to those relevant under climate change scenarios. Such assessment is essential to predict the performance of this species of high ecological relevance in Arctic fjord systems.

Hence, this study aims to explore the effect of interacting environmental drivers relevant to the habitat of the brown seaweed *D. aculeata*, specifically salinity fluctuations and light cycles (irradiance) at low temperatures. In addition, the study will contribute to our understanding of the limits of physiological tolerance of *D. aculeata* from the Arctic in a scenario of climate change.

## 2. Materials and Methods

### 2.1. Collection of Algal Material

This experiment was conducted at Kings Bay Marine Laboratory, Ny-Ålesund, Kongsfjorden, Spitsbergen-Svalbard in July 2019. Samples of the brown seaweed *Desmarestia aculeata* were collected in the intertidal zone at low tide in front of the marine laboratory (78°55′39.8″ N; 11°55′48.3″ E). The specimens were kept in seawater while they were cleaned from epiphytes and sediment. Subsequently, samples of algal tissue were collected from the frond.

### 2.2. Experimental Set-Up

The samples were kept in a pre-control treatment, for five days, in aerated 1 L tanks, at 0 °C with salinity (S_A_) 34 seawater enriched with 1/2 Provasoli solution (1/2 PES, [29], modifications: HEPES-buffer instead of Tris, double the concentration of Na_2_ glycerophosphate, iodine enrichment after [30]) and a constant irradiance of 50 μmol photons m^−2^s^−1^. After an acclimation period over 5 days, the experiment ran for 21 days. Therefore, the samples were pre-acclimatised to the control salinity S_A_ 34. For 21 days at the start of the experiment, a part of the samples was kept at the control S_A_ 34 and we proceeded to test the effects of hyposalinity at S_A_ 28 and 18. All treatments were maintained at 0 °C. To simulate the meltwater inflow from glacial run-off, the water in the experiment was diluted using fresh water [31]. The water was exchanged every fourth day throughout the experiment. Measurements and samples were taken on days 1 and 21.

Regarding the light intensity values, these are based on Kongsfjorden values during a daily cycle [32]. Irradiance was cycled every 12 h between the highest irradiance point at 500 μmol photons m^−2^s^−1^ (HL) and the lowest at 50 μmol photons m^−2^s^−1^ (LL). The light points were reached every 12 h, increasing hourly for 12 h until HL was reached and decreasing hourly for 12 h until LL was reached, making up the 24 h daily cyclic irradiance. Measurements were made specifically at 500 μmol photons m^−2^s^−1^ (HL) and at the 50 μmol photons m^−2^s^−1^ (LL) period. The configuration of the light cycles was carried out using the ProfiLux 3 system (with LED Mitras daylight, GHL Advanced Technology, Kaiserslautern, Germany).

After the measurements (see below Physiological parameters), the samples were shock-frozen in liquid nitrogen and stored at −80 °C. Afterward, the samples were freeze-dried for 24 h, the dry weight (DW) was obtained, and the biochemical analyses were performed.

### 2.3. Physiological Parameters

The maximum quantum yield of photosystem II (*F*_v_/*F*_m_) was measured in vivo after leaving the samples for 10 min in the dark. Subsequently, photosynthesis-irradiance (P-E) curves were recorded up to an irradiance of 600 μmol photons m^−2^s^−1^ to obtain the following parameters: photosynthetic capacity expressed as maximum relative electron transport rate (rETRmax), saturation irradiance (*E*k), and photosynthetic efficiency (α, initial linear slope). The (P-E) curves were fitted according to the equation of Platt et al. [33], using the program KaleidaGraph version 4.5.4 (Synergy Software, Reading, PA, USA). All analyses were performed using an amplitude-modulated chlorophyll fluorometer (Imaging PAM, Heinz Walz GmbH, Effeltrich, Germany).

### 2.4. Biochemical Parameters

Pigment analysis of *Desmarestia aculeata* was carried out following the methodology of Koch et al. [34] for brown seaweeds. Freeze-dried samples weighing 30 mg (n = 3) were measured using a high-performance liquid chromatography (HPLC) LaChromeElite system with a chilled L-2200 autosampler and an L-2450 DAD detector (VWR-Hitachi International GMBh, Darmstadt, Germany). Subsequently, using the methodology of Wright et al. [35], the following pigments were quantified: Chlorophyll *a* (Chl *a*), Chlorophyll *c2* (Chl *c2*), Fucoxanthin (Fucox), β-Carotene (β-Car), Violaxanthin (Viol), Antheraxanthin (Anthera) and Zeaxanthin (Zeax). Pigment content was finally expressed as µg g^−1^ (DW). Additionally, the xanthophyll cycle pool (VAZ: violaxanthin, antheraxanthin, zeaxanthin) and the de-epoxidation state (DPS) were calculated for *D. aculeata*.

The quantification of the content of the sugar alcohol mannitol in *Desmarestia aculeata* was carried out following the methodology proposed by Karsten et al. [36]. Freeze-dried samples weighing 10–15 mg were incubated in 1 mL of aqueous ethanol (70%, *v*/*v*) in a water bath at 70 °C for 4 h. Concentration determination was performed according to Diehl et al. [26]. D(-)-mannitol (C_6_H_14_O_6_, Roth) standards of 1, 10, and 20 mM were used for calibration. The mannitol concentration was expressed in mg g^−1^ (DW).

For total carbon C (% DW), total nitrogen N (% DW), and C:N (%) ratio, 4–5 mg of freeze-dried sample (n = 3) sampled at days 1 and 21 of the *Desmarestia aculeata* culture were used. The measurement time of each sample was 150 s The samples were combusted at 1000 °C, acetanilide (C_8_H_9_NO) was used as standard, C and N samples were quantified using the Euro EA 3000 Elemental Analyser (Eurovector S.P.A., Milan, Italy). Total C and N concentrations were expressed in mg g^−1^ dry weight (DW). The C:N ratio (%) was obtained based on these results.

The concentration of phlorotannins in *D. aculeata* was measured using the Folin-Ciocalteu method described by Cruces et al. [37]. Purified phloroglucinol (Sigma-Aldrich) was used as standard. Freeze-dried samples of 20 mg (n = 3) were used for extraction and quantification. One millilitre of acetone (70%, *v*/*v*) was added to each sample and subsequently kept at 4 °C for 24 h in the dark. Absorbance was measured at λ = 730 nm using a microplate spectrophotometer. Finally, the quantification of total soluble phlorotannins was expressed in mg g^−1^ (DW).

### 2.5. Statistical Analysis

Statistical analyses were performed considering day 1 and 21 of culture. Tests for normal distribution (Shapiro-Wilk test; *p* > 0.05) were performed for all data sets. The data were log_10_-transformed where necessary. Three-way ANOVAs were then performed for each parameter measured. Tukey’s post-hoc test was applied to detect significant differences (*p* < 0.05). This test was applied for each parameter analysed. Statistical analyses were run using RStudio (version 1.1.383, Boston, MA, USA).

## 3. Results

Regarding the photosynthetic parameters of *Desmarestia aculeata*, only a few clear impacts could be determined (Table 1). The rETRmax and α tended to decrease over time, while *E*k and the *F*_v_/*F*_m_ remained almost unchanged. Even though significant differences regarding salinity were found for rETRmax and *E*k. Light had no major impact on the photosynthesis, however, significantly higher rETRmax, *E*k and *F*_v_/*F*_m_ values were measured in the HL treatments, mainly at S_A_ 34 on day 1. Interestingly, different interactions between days, light and salinity were detected within all parameters, mainly in the parameters rETRmax, *E*k, and *F*_v_/*F*_m_ (Table S1: Photosynthetic parameter).

Pigments were almost unaffected throughout the experiment and by the different treatments (Table 2 and Table 3), and an interaction between days, light and salinity was only detected for fucoxanthin (Table S1: Pigments). Few significant differences in pigment concentrations were found for Chl *c2*, β-Car and Fucox, which, however, could not be directly assigned to the sampling day, high light (HL), low light (LL) or hyposalinity. The VAZ significantly decreased from day 1 to day 21 at S_A_ 34 and 28, and revealed lower concentrations in the HL treatments on day 1. Increases in the DPS were only determined when comparing day 1 and 21 at S_A_ 34 and 28. No differences were found for the light and salinity treatments.

**Figure 1:**
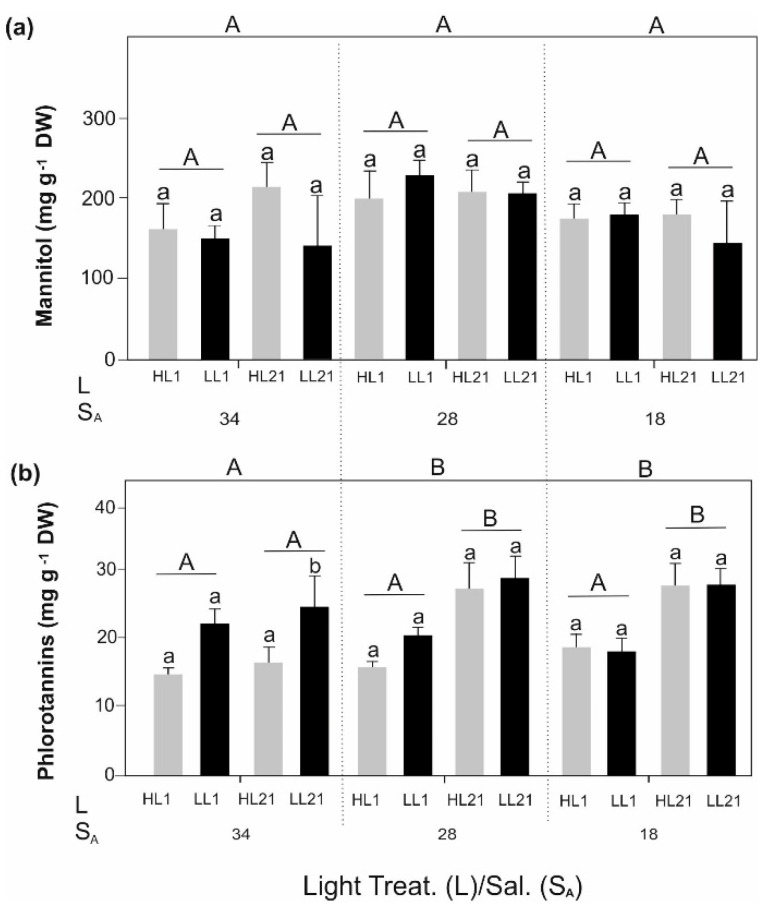
Concentration in mg g^−1^ dry weight (DW) for compounds in *Desmarestia aculeata*: (**a**) Mannitol; (**b**) Phlorotannins. Experimental set-up: salinity (S_A_)–Light (L) (High Light (HL)–Low Light (LL)) treatments. Values are means ± SD (n = 3). Statistically significant differences between HL and LL per treatments are marked by different lowercase letters. Statistically significant differences between treatment days (1–21) are marked by different uppercase letters and differences between salinities (S_A_ 34, 28, and 18) are marked by different uppercase letters outside the graph. For all the data, three-way ANOVA with post-hoc Tukey’s test was performed (*p* < 0.05).

Mannitol (Figure 1a) neither changed over time nor was affected by light or salinity. However, significant interactions between light and salinity were found (Table S1: Sugar Alcohol). Overall, high mannitol values were recorded in all samples during the experiment (Figure 1a). Significantly higher phlorotannin concentrations were measured at lower salinities (S_A_ 28 and 18) and the content increased over time (Figure 1b). Regarding the different light treatments, no significant differences between HL and LL were detected, except for the samples from day 21 at S_A_ 34.

**Figure 2:**
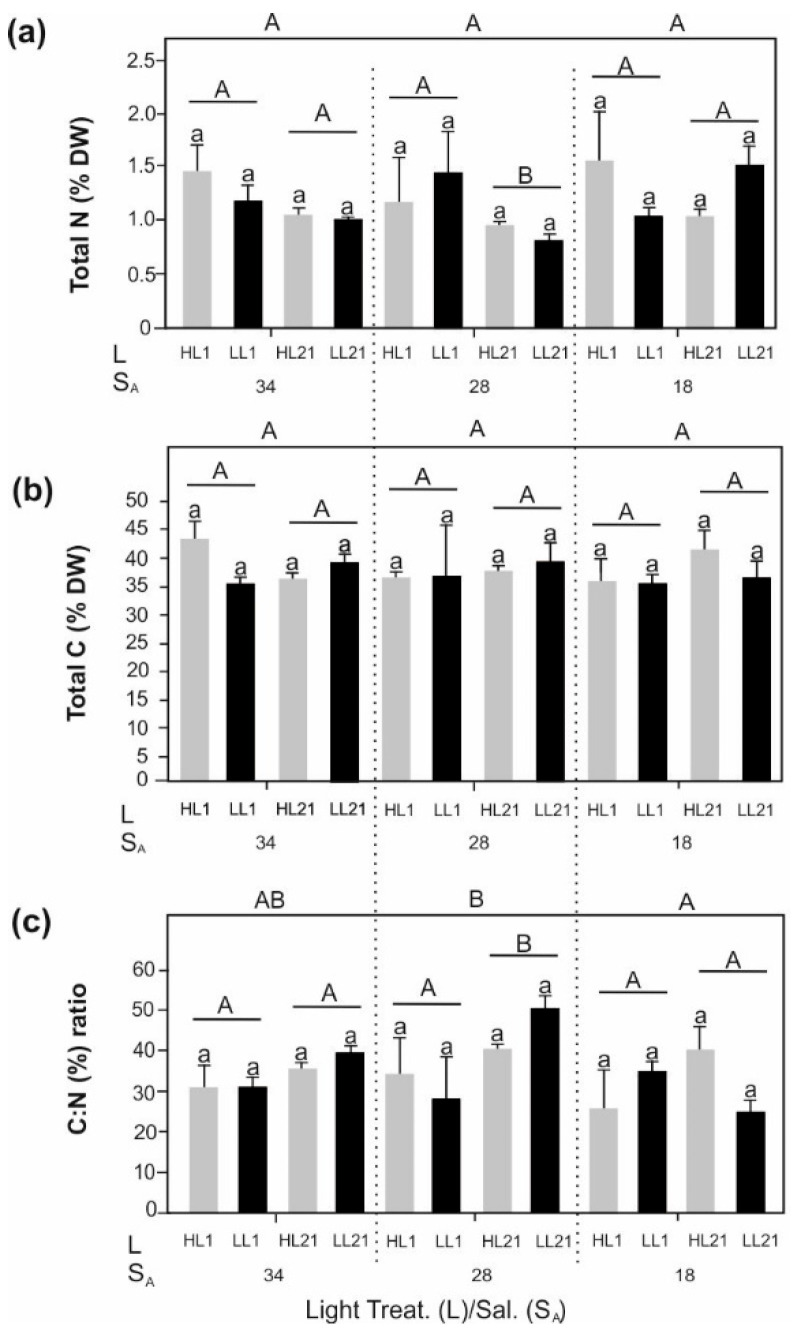
Elemental composition of *Desmarestia aculeata* at days 1 and 21 of treatment. (**a**) Contents of total N (% DW); (**b**) Contents of total C (% DW); (**c**) C:N ratio. Experimental set–up: salinity (S_A_)—Light (L) (High Light (HL)–Low Light (LL)) treatments. Values are means ± SD (n = 3). Statistically significant differences between HL and LL per treatments are marked by different lowercase letters. Statistically significant differences between treatment days (1–21) are marked by different uppercase letters and differences between salinities (S_A_ 34, 28, and 18) are marked by different uppercase letters outside the graph. For all the data, three-way ANOVA with post-hoc Tukey’s test was performed (*p* < 0.05).

The total carbon (C) content remained completely unchanged throughout the experiment (Figure 2b). Similarly, total nitrogen (N) showed no significant differences with light or salinity (Figure 2a). However, total N significantly changed over time; there was no trend towards higher or lower concentrations. The C:N ratio did not reveal clear effects in the different treatments (Figure 2c). Over time, higher C:N ratios were only found at S_A_ 28 and the significant impact by salinity could not be assigned to the absolute salinities in general. Significantly lower total N at S_A_ 28 and HL on days 21 resulted in significantly higher C:N ratios in the same samples. However, total N, total C and the C:N ratio exhibited days:light:salinity interactions (Table S1: Total Contents).

## 4. Discussion

In this study, physiological and biochemical acclimation processes of the brown seaweed *Desmarestia aculeata* were evaluated after simulating different salinity conditions and diurnal changes in irradiance. The tested environmental parameters and time were observed to mainly have an impact on the photosynthetic responses of *D. aculeata*, while biochemical acclimation was less pronounced. The observed strong interactive effects of light, salinity and time highlight the complex interplay of the various environmental factors affecting the species.

Salinity variation, as a consequence of increased meltwater discharge to Arctic fjords, has been widely described as a direct effect of climate change [38,39]. It has been observed that decreasing salinity in the first few meters of the water column directly affects photosynthetic performance in polar brown algae [26,27,40]. However, in addition to such changes occurring by global warming, diurnal variation in irradiance represents an additional stress factor for seaweeds. Hanelt et al. [41,42] described how fluctuations in daily irradiance levels during the Arctic summer affect internal photosynthetic and biochemical regulation in seaweeds. Hence, brown seaweeds inhabiting the shallow subtidal zone are constantly exposed to marked variations in salinity and irradiance levels. Our study revealed the resilience of the species *D. aculeata* to environmental changes, namely changes in salinity and irradiance, facilitated by a high plasticity of its internal regulation.

This high tolerance of certain brown seaweeds to varying salinity agrees with what was recorded in our experiment. The *F*_v_/*F*_m_ of *D. aculeata* was not diminished by decreasing salinities. However, other photosynthetic parameters such as rETRmax and *E*k showed clear responses to low salinities of S_A_ 28 and 18, respectively. The variations of rETRmax and *E*k show how this species is able to regulate its photosynthetic activity and ensure that the maximal quantum yield of PSII is maintained at high values. Irradiance plays a fundamental role in the photosynthetic processes of seaweeds [43]. However, during our experiment, the photosynthetic variables of *D. aculeata* were not generally affected by variations in the daily course of irradiance. The fact that both high light (HL) and low light (LL) had no major effect highlights the high plasticity of *D. aculeata* to a fluctuating light climate, given by its ability for internal regulation. This observation differs from the high sensitivity described for *D. aculeata* under constant light intensities. Marambio and Bischof [24] described how high constant irradiance affects the photosynthetic parameters of *D. aculeata*, such as *F*_v_/*F*_m_, rETRmax, α, and *E*k, over time. *F*_v_/*F*_m_ has been observed to decrease during periods of high irradiance, for example in *L. digitata* and *Saccharina latissima* [44,45] or *Chondrus crispus* analysed under natural and laboratory conditions [46]. All these species showed a high acclimation to daily cyclic variations in irradiance.

Regarding pigments, we observed that Chl *a* and β-Car in *D. aculeata* were neither affected by high nor low daily irradiance, or by low salinity. On the other hand, the accessory pigments Chl *c2* and Fucox apparently have a crucial role as photosynthetic regulators: under the variation of daily cyclic irradiance, these two pigments showed a high acclimation to HL and LL during the experiment at high and low salinity, but VAZ and DPS were not affected by the low salinities S_A_ 28 and 18. This is contrary to what has been observed in other species, such as *S. latissima*, which in laboratory culture has been shown to be strongly affected by the low salinity [47].

Mannitol is part of carbon storage in the photosynthetic process in brown seaweeds [48]. Additionally, this photosynthetic product acts as protectant against osmotic stress [49,50]. However, during our experiment, we did not find any effect of salinity or irradiance on the mannitol concentration in *D. aculeata* during the 21 days of treatment. Still, high concentrations were observed throughout all treatments. On the one hand, constant high mannitol content could be an acclimation to the frequently experienced environmental fluctuations in the intertidal zone, where *D. aculeata* was collected. On the other hand, we suspect that the high concentrations are an additional thermal protection mechanism of *D. aculeata*, since mannitol can also act as an anti-freezing compound [51]. As reported by Monteiro et al. [52], the brown alga *S. latissima* also reaches concentrations of approximately 200 mg g^−1^ DW at S_A_ 30 and 0 °C, which is in agreement with what was measured in our study. However, the effect of low temperature on *D. aculeata* was not specifically evaluated during our experiment.

Phlorotannins, another important group of brown algal compounds, have been described as contributing to the reinforcement of cell walls under hyposaline conditions, e.g., in the brown alga *Alaria esculenta* [53]. In addition, phlorotannins are actively involved in protection against intense irradiance, and protection of tissues against pathogenic microbial activity and herbivory [54,55,56]. For *D. aculeata,* high phlorotannin values were observed at all salinities tested in this experiment. Our results are in agreement with those described by Springer et al. [53] for *A. esculenta* at different salinity levels and with those observed by Ragan & Jensen [57] for *Fucus vesiculosus* during winter with low temperatures. Although the content of phlorotannins is overall high, a variation in concentration was observed at S_A_ 34 under daily cycles of irradiance. Importantly, phlorotannins may be activated depending on conditions and move through the cell wall to increase their site-specific content [53,58]. It remains unresolved whether the response of phlorotannins to S_A_ 28 and 18 on day 21 of cultivation is a driver-specific or a non-specific response of the seaweed to non-favourable conditions.

In contrast to previous studies on *L. solidungula* [34] and *F. serratus* [59] from Spitsbergen, total N and the C:N ratio of *D. aculeata* were not affected by salinity variations. Samples were also not affected by HL and LL. However, even though the samples were maintained at ½ PES, all treatments revealed C:N ratios higher than 20, indicating N limitation [60]. The uptake and assimilation of nutrients are known to be impacted by temperature-changing enzymatic processes [61]. It is possible that N limitation was caused by reduced N uptake potential due to the slowed enzymatic action at 0 °C.

## 5. Conclusions

The response of this population of *D. aculeata* revealed a high potential for acclimation to the different environmental parameters to which it was exposed to. In our experiment, *D. aculeata* responded rapidly through metabolic regulation to cyclic light variations and was not affected by the hyposaline condition. As previously mentioned, the *D. aculeata* population studied inhabits the intertidal and upper subtidal zone and is therefore constantly exposed to a highly dynamic abiotic environment, including exposure to meltwater (field observation). This could explain the high plasticity of this population of *D. aculeata* to low salinity and large fluctuations in irradiance. The reaction of photosynthetic and biochemical parameters of *D. aculeata* to different factors shows how this phenotype can cope with change in multiple drivers. This mechanism is key to generating resistance through a rapid and effective acclimation process [40,62].

Ecologically, *D. aculeata* is not a strictly polar seaweed; its life history as a temperate-boreal species gives it a unique characteristic, and it is distributed over a wide geographical range. Therefore, future changes in the habitat of this species will be determined by the intensity and duration of climate change events. Our data support that this population of *D. aculeata* will be able to quickly adjust to changing environmental conditions in the Arctic coastal zone due to its high ecophysiological plasticity. Finally, through the study of this population, it was possible to obtain important information about the resilience of these *D. aculeata* individuals, in order to predict future responses to environmental changes.

## Figures and Tables

**Table 1:** Photosynthetic parameters of *Desmarestia aculeata*: rETRmax, α, *E*k, and *F*_v_/*F*_m_. Experimental set-up: salinity (S_A_)–Light (L) (High Light (HL)–Low Light (LL)) treatments. Values are means ± SD (n = 3). For each parameter (L, Days, S_A_), statistically significant differences are marked by different lowercase letters. For all the data, three-way ANOVA with post-hoc Tukey’s test was performed (*p* < 0.05).

S_A_	Days	L	rETRmax	Significance		α		Significance		*E*k		Significance			*F*_v_/*F*_m_	Significance		
			(rel. Units)	L	Days	S_A_	(µmol Photons m^−2^s^−1^)^−1^	L	Days	S_A_	(µmol Photons m^−2^s^−1^)	L	Days	S_A_	(rel. Units)	L	Days	S_A_
34	1	HL	31.360 (±2.05)	a	a	ab	0.202 (±0.01)	a	a	a	156.473 (±22.28)	a	a	a	0.618 (±0.01)	a	a	a
		LL	20.183 (±2.78)	b			0.213 (±0.01)	a			94.597 (±8.38)	b			0.427 (±0.10)	b		
	21	HL	13.130 (±1.77)	a	b		0.193 (±0.02)	a	a		69.221 (±17.52)	a	b		0.399 (±0.10)	a	a	
		LL	12.893 (±1.25)	a			0.229 (±0.02)	a			56.430 (±6.35)	a			0.532 (±0.02)	a		
28	1	HL	18.557 (±3.12)	a	a	a	0.235 (±0.03)	a	a	a	79.370 (±12.64)	a	a	b	0.613 (±0.06)	a	a	a
		LL	20.656 (±2.12)	a			0.256 (±0.02)	a			80.903 (±7.68)	a			0.590 (±0.06)	a		
	21	HL	13.046 (±0.52)	a	b		0.202 (±0.02)	a	b		64.996 (±7.43)	a	a		0.511 (±0.03)	a	a	
		LL	14.239 (±2.14)	a			0.187 (±0.02)	a			76.627 (±14.93)	a			0.394 (±0.04)	b		
18	1	HL	22.529 (±3.42)	a	a	b	0.233 (±0.02)	a	a	a	97.819 (±22.09)	a	a	a	0.498 (±0.11)	a	a	a
		LL	21.794 (±5.33)	a			0.201 (±0.04)	a			110.722 (±29.33)	a			0.359 (±0.05)	a		
	21	HL	20.863 (±4.95)	a	a		0.173 (±0.02)	a	b		120.807 (±24.36)	a	a		0.512 (±0.03)	a	a	
		LL	16.210 (±3.97)	a			0.178 (±0.02)	a			90.114 (±13.74)	a			0.532 (±0.03)	a		

**Table 2:** Concentration of main pigments [µg g^−1^ dry weight (DW)] of *Desmarestia aculeata*: Chl *a*, Chl *c2*, β-Car, Fucox. Experimental set-up: salinity (S_A_)–Light (L) (High Light (HL)–Low Light (LL)) treatments. Values are means ± SD (n = 3). For each parameter (L, Days, S_A_), statistically significant differences are marked by different lowercase letters. For all the data, three-way ANOVA with post-hoc Tukey’s test was performed (*p* < 0.05).

S_A_	Days	L	Chl *a*	Significance			Chl *c2*	Significance			β-Car	Significance			Fucox	Significance		
			(µg g^−1^ DW)	L	Days	S_A_	(µg g^−1^ DW)	L	Days	S_A_	(µg g^−1^ DW)	L	Days	S_A_	(µg g^−1^ DW)	LL	Days	S_A_
34	1	HL	478.73 (±61.74)	a	a	a	60.60 (±15.42)	a	a	ab	21.03 (±4.32)	a	a	a	178.83 (±6.48)	a	a	a
		LL	450.60 (±44.34)	a			51.07 (±5.52)	a			17.07 (±3.76)	a			192.60 (±6.24)	a		
	21	HL	447.53 (±51.20)	a	a		58.10 (±9.40)	a	a		18.10 (±2.07)	a	a		203.63 (±27.75)	a	a	
		LL	328.70 (±41.66)	a			87.73 (± 3.25)	a			12.03 (±1.68)	a			140.93 (±28.62)	a		
28	1	HL	526.90 (±144.48)	a	a	a	85.60 (±11.82)	a	a	a	19.57 (±5.86)	a	a	a	177.47 (±9.46)	a	a	a
		LL	536.83 (±192.22)	a			62.53 (±4.83)	a			21.10 (±7.95)	a			144.10 (±1.73)	a		
	21	HL	394.53 (±74.28)	a	a		53.87(±14.77)	a	a		11.37 (±2.94)	a	b		183.17 (±43.07)	a	a	
		LL	391.57 (±33.67)	a			70.63 (±3.40)	a			11.07 (±0.67)	a			163.97 (±13.98)	a		
18	1	HL	454.47 (±111.25)	a	a	a	68.80 (±2.51)	a	a	b	16.03 (±1.44)	a	a	a	167.43 (±6.82)	a	a	a
		LL	405.10 (±12.87)	a			48.37 (±2.32)	b			15.17 (±1.22)	a			165.27 (±2.25)	a		
	21	HL	334.97 (±43.70)	a	a		39.37 (±5.89)	a	a		15.10 (±0.50)	a	a		140.03 (±20.06)	a	a	
		LL	472.90 (±33.20)	a			76.77 (±6.62)	b			11.00 (±3.80)	a			228.37 (±15.87)	b		

**Table 3:** The pool of the xanthophyll cycle—VAZ (µg g^−1^ dry weight (DW)) and de-epoxidation state (DPS). Experimental set–up: salinity (S_A_)–Light (L) (High Light (HL)–Low Light (LL)) treatments of *Desmarestia aculeata*. Values are means ± SD (n = 3). For each parameter (L, Days, S_A_), statistically significant differences are marked by different lowercase letters. For all the data, three-way ANOVA with post-hoc Tukey’s test was performed (*p* < 0.05).

S_A_	Days	L	VAZ	Significance			DPS	Significance		
			(µg g^−1^ DW)	L	Days	S_A_		L	Days	S_A_
34	1	HL	0.35 (±0.03)	a	a	a	1.58 (±0.12)	a	a	a
		LL	0.27 (±0.04)	a			1.95 (±0.28)	a		
	21	HL	0.26 (±0.02)	a	b		2.16 (±0.07)	a	b	
		LL	0.21 (±0.03)	a			2.70 (±0.32)	a		
28	1	HL	0.40 (±0.06)	a	a	a	1.48 (±0.26)	a	a	a
		LL	0.29 (±0.08)	b			2.01 (±0.66)	a		
	21	HL	0.23 (±0.04)	a	b		2.44 (±0.37)	a	b	
		LL	0.19 (±0.04)	a			2.82 (±0.41)	a		
18	1	HL	0.29 (±0.02)	a	a	a	1.83 (±0.15)	a	a	a
		LL	0.24 (±0.02)	b			2.26 (±0.17)	a		
	21	HL	0.22 (±0.03)	a	a		2.52 (±0.31)	a	a	
		LL	0.26 (±0.01)	a			2.17 (±0.13)	a		

## Data Availability

Data are available in PANGEA.

## Supplementary Materials

The following supporting information can be downloaded at: https://www.mdpi.com/article/10.3390/biology11101499/s1. Table S1: Results of three-way ANOVA for *Desmarestia aculeata*.
