# Supplementary material for: High Ecophysiological Plasticity of Desmarestia aculeata (Phaeophyceae) Present in an Arctic Fjord under Varying Salinity and Irradiance Conditions
Source: Biology (Basel). 2022 Oct 13;11(10):1499. doi: 10.3390/biology11101499 (PMC9598539; doi:10.3390/biology11101499)
Supplement: Supplementary file 1 [file biology-11-01499-s001.zip › biology-1910806-supplementary.pdf]

Supplementary Materials:

Table S1.

Results of three-way ANOVA for *Desmarestia aculeata* (n=3): effects of the daily cyclic irradiance and salinities. Photosynthetic parameters (F_v/F_m, α, *E*k, and rETRmax); Pigments (Chl *a*, Chl *c2*, β-Car, Fucox, VAZ, and DPS); Total elemental contents (C %, N %, and C:N % ratio); Phlorotannins; Mannitol. Non-parametric data were transformed and are marked by (•). Asterisks (*) indicate statistically significant values: (***) p<0.001, (**) p<0.01, and (*) p<0.05.

	Variable	Factor	*df*	*F*-value	*p*-value	
Photosynthetic parameter	**rETRmax**	Days	1	51.620	<0.001	***
		Light	1	4.716	0.040	*
		Salinity	2	4.639	0.020	*
		Days:Light	1	0.967	0.335	
		Days:Salinity	2	6.982	0.004	**
		Light:Salinity	2	4.237	0.027	*
		Days:Light:Salinity	2	4.783	0.018	*
	***E*k**	Days	1	17.007	<0.001	***
		Light	1	5.326	0.030	*
		Salinity	2	9.000	0.001	**
		Days:Light	1	0.206	0.654	
		Days:Salinity	2	11.933	<0.001	***
		Light:Salinity	2	5.044	0.015	*
		Days:Light:Salinity	2	5.503	0.011	*
	**α**	Days	1	15.524	<0.001	***
		Light	1	0.347	0.561	
		Salinity	2	3.300	0.054	
		Days:Light	1	0.352	0.558	
		Days:Salinity	2	5.101	0.014	*
		Light:Salinity	2	1.979	0.160	
		Days:Light:Salinity	2	2.282	0.124	
	**F_v/F_m**	Days	1	3.283	0.083	
		Light	1	6.512	0.018	*
		Salinity	2	2.132	0.141	
		Days:Light	1	9.735	0.005	**
		Days:Salinity	2	11.617	<0.001	***
		Light:Salinity	2	0.360	0.702	

		Days:Light:Salinity	2	8.615	0.002	**
Pigments	**Chl *a***	Days	1	3.215	0.058	
		Light	1	0.088	0.769	
		Salinity	2	0.924	0.411	
		Days:Light	1	0.233	0.624	
		Days:Salinity	2	1.278	0.297	
		Light:Salinity	2	1.428	0.260	
		Days:Light:Salinity	2	2.053	0.150	
	**Chl *c2***	Days	1	3.341	0.080	
		Light	1	0.319	0.578	
		Salinity	2	4.168	0.028	*
		Days:Light	1	66.083	<0.001	***
		Days:Salinity	2	2.203	0.132	
		Light:Salinity	2	8.984	0.001	**
		Days:Light:Salinity	2	1.187	0.322	
	**β-Car**	Days	1	17.822	<0.001	***
		Light	1	3.448	0.076	
		Salinity	2	1.633	0.216	
		Days:Light	1	0.934	0.343	
		Days:Salinity	2	2.602	0.095	
		Light:Salinity	2	1.738	0.197	
		Days:Light:Salinity	2	0.030	0.970	
	**Fucox**	Days	1	0.786	0.384	
		Light	1	0.156	0.696	
		Salinity	2	0.165	0.329	
		Days:Light	1	0.528	0.475	
		Days:Salinity	2	2.246	0.128	
		Light:Salinity	2	12.451	<0.001	***
		Days:Light:Salinity	2	13.914	<0.001	***
	**VAZ**	Days	1	31.556	<0.001	***
		Light	1	13.284	0.001	**
		Salinity	2	1.216	0.314	
		Days:Light	1	5.056	0.340	
		Days:Salinity	2	5.300	0.124	
		Light:Salinity	2	2.709	0.087	
		Days:Light:Salinity	2	0.472	0.630	
	**DPS**	Days	1	35.310	<0.001	***

		Light	1	3.256	0.054	
		Salinity	2	0.356	0.704	
		Days:Light	1	1.394	0.249	
		Days:Salinity	2	2.667	0.090	
		Light:Salinity	2	1.813	0.185	
		Days:Light:Salinity	2	1.840	0.181	
Sugar Alcohol	**Mannitol•**	Days	1	0.845	0.367	
		Light	1	0.136	0.715	
		Salinity	2	0.226	0.799	
		Days:Light	1	0.113	0.739	
		Days:Salinity	2	0.536	0.592	
		Light:Salinity	2	7.914	0.002	**
		Days:Light:Salinity	2	3.381	0.051	
Phlorotannins	**Phloro•**	Days	1	64.722	<0.001	***
		Light	1	16.132	<0.001	***
		Salinity	2	6.828	0.004	**
		Days:Light	1	0.038	0.847	
		Days:Salinity	2	8.537	0.002	**
		Light:Salinity	2	6.386	0.006	**
		Days:Light:Salinity	2	0.569	0.573	
Total Contents	**N (%)•**	Days	1	12.742	0.002	**
		Light	1	0.385	0.541	
		Salinity	2	3.215	0.058	
		Days:Light	1	2.346	0.139	
		Days:Salinity	2	3.710	0.039	*
		Light:Salinity	2	0.547	0.586	
		Days:Light:Salinity	2	8.760	0.001	**
	**C (%)•**	Days	1	0.748	0.396	
		Light	1	1.575	0.222	
		Salinity	2	0.422	0.661	
		Days:Light	1	1.203	0.284	
		Days:Salinity	2	1.744	0.196	
		Light:Salinity	2	1.315	0.287	
		Days:Light:Salinity	2	4.013	0.031	*
	**C:N (%)**	Days	1	15.109	<0.001	***
		Light	1	0.010	0.920	
		Salinity	2	3.649	0.041	*
		Days:Light	1	0.178	0.677	

Days:Salinity	2	2.971	0.070	
Light:Salinity	2	0.641	0.536	
Days:Light:Salinity	2	9.755	<0.001	***

(•) log_{10} transformation
